# Recommendations for contouring of gross tumour volume for locally advanced lung cancer using magnetic resonance imaging

**DOI:** 10.1016/j.phro.2026.100948

**Published:** 2026-03-15

**Authors:** Anna-Maria Shiarli, Michael J. Dubec, Merina Ahmed, Jon T. Asmussen, Hannah Bainbridge, José S. Belderbos, Sean Brown, Johan Bussink, David Cobben, Bram H.J. Geurts, Andrew Hope, John Kavanagh, Dow-Mu Koh, Ferry Lalezari, Alexander V. Louie, Laura G. Merckel, Firdaus A.M. Hoesein, James P.B. O’Connor, Rocio Perez-Johnston, Tyson J. Reeve, Andreas Rimner, Peter S.N. van Rossum, Dominic A.X. Schinagl, Tine Schytte, Craig Stevens, Alex Tan, Rob H.N. Tijssen, Nina Tunariu, Marcel Van Herk, Joost J.C. Verhoeff, Andreas Wetscherek, Rianne Wittenberg, David Woolf, Corinne Faivre-Finn, Fiona McDonald

**Affiliations:** a The Institute of Cancer Research, London, UK; b The Royal Marsden NHS Foundation Trust, London, UK; c Cambridge University Hospitals NHS Foundation Trust, Cambridge, UK; d Division of Cancer Sciences, Faculty of Biology, Medicine and Health, University of Manchester, Manchester, UK; e The Christie NHS Foundation Trust, Manchester, UK; f Department of Radiology Odense University Hospital, Odense, Denmark; g Department of Radiotherapy, Portsmouth University Hospital, Portsmouth, UK; h Department of Radiation Oncology, The Netherlands Cancer Institute, Amsterdam, the Netherlands; i Gloucestershire Oncology Centre, Gloucestershire Hospitals NHS Foundation Trust, UK; j Radboud University Medical Centre, Nijmegen, the Netherlands; k Department of Health Data Science, Institute of Population Health, University of Liverpool, Liverpool, UK; l The Clatterbridge Cancer Centre NHS Foundation Trust, Liverpool, UK; m Radiation Medicine Program, Princess Margaret Cancer Centre, University Health Network, Toronto, Ontario, Canada; n Department of Radiation Oncology, University of Toronto, Toronto, Ontario, Canada; o Cardiothoracic Imaging, Toronto Joint Department of Medical Imaging, University Health Network, Toronto, Ontario, Canada; p Department of Radiology, The Netherlands Cancer Institute/Antoni van Leeuwenhoek, Amsterdam, the Netherlands; q Department of Radiation Oncology, Odette Cancer Centre, Sunnybrook Health Sciences Centre, Toronto, Canada; r Department of Radiotherapy, University Medical Center Utrecht, Utrecht, the Netherlands; s Department of Radiology, University Medical Center Utrecht, Utrecht, the Netherlands; t Memorial Sloan Kettering Cancer Center, New York, United States; u Queensland XRay, Townsville, Queensland, Australia; v Department of Oncology, Odense University Hospital, Odense, Denmark; w Department of Clinical Research, University of Southern Denmark, Odense, Denmark; x Department of Radiation Oncology, Beaumont Health, MI, United States; y Sunshine Coast University Hospital, Queensland, Australia; z James Cook University School of Medicine and Dentistry, Queensland, Australia; aa Department of Radiation Oncology, Catharina Hospital, Eindhoven, the Netherlands; ab Joint Department of Physics, The Institute of Cancer Research and The Royal Marsden NHS Foundation Trust, London, UK

**Keywords:** Thoracic MRI, Lung cancer, Radiotherapy

## Abstract

**Background and purpose:**

The use of Magnetic Resonance imaging (MRI) for radiotherapy planning and guidance for locally advanced non-small cell lung cancer (LA NSCLC) is novel. The superior soft tissue definition of MRI compared to CT, may facilitate more accurate gross tumour volume (GTV) definition, with the goal of improving radiotherapy precision. This work aims to develop GTV contouring recommendations for NSCLC on MRI.

**Materials and methods:**

Two international training workshops on GTV delineation for LA NSCLC were attended by thoracic radiation oncologists and MR radiologists. Thoracic radiation oncology experts contoured nine cases of LA NSCLC, firstly, on mid-position 4D-CT with PET-CT guidance, and secondly on non-contrast MRI, registered with the CT and PET-CT. Consensus contours generated on CT and MRI were discussed and finalised during two international meetings.

**Results:**

Recommendations on GTV delineation for LA NSCLC for both the primary tumour and individual lymph node stations using thoracic MRI were produced and are provided in this document. Consensus contours generated on CT and MRI for specific clinical scenarios were demonstrated and challenges addressed.

**Conclusions:**

We provide the first set of consensus recommendations on GTV contouring on MRI for LA NSCLC through an international collaborative process between international experts in thoracic radiation oncology and MR radiology. This work provides an initial step towards standardisation of lung GTV delineation on MRI, which is necessary prior to any meaningful assessment of the benefits of MRI in GTV definition compared to current practice.

## Highlights

• Recommendations on GTV contouring for LA NSCLC using MRI are provided.
• Recommendations developed by panel from multiple international institutions.
• Challenging areas included tumour invasion into chest wall and pulmonary vessels.
• MRI interpretation and contouring of thoracic lymph nodes is provided.

## Graphical abstract

**Figure:**
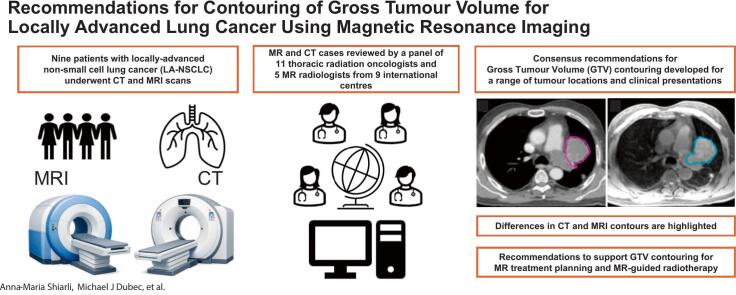

## 1 Introduction

Unresectable locally advanced non-small cell lung cancer (LA NSCLC) is radically treated with chemoradiotherapy (CRT) or radiotherapy (RT). The superior soft tissue contrast of magnetic resonance imaging (MRI) is currently used only in NSCLC staging, to define tumour thoracic inlet and chest wall invasion, and brachial plexus involvement [1], [2], [3], [4], [5], [6]. However, as with the incorporation of 18-fluorodeoxyglucose positron emission tomography – computed tomography (^18^F-FDG PET-CT) [7], [8], [9], the use of MRI information may improve Gross Tumour Volume (GTV) definition for NSCLC, at the RT planning stage and within MRI-guided RT (MRgRT) workflows [10], [11]. MRI NSCLC GTV contouring guidelines do not currently exist but have the potential to improve delineation precision [12], [13], [14], [15].

Since MRI is not used routinely in RT planning for lung cancer, clinician education on MRI interpretation, and GTV contouring guidance, is crucial, before studies can assess improvements in GTV contouring accuracy. Factors including the low proton density in the lung, magnetic susceptibility effects, and motion, affect thoracic MRI image quality [16], [17], [18] making MRI interpretation challenging. The aim of this work was to develop recommendations for MRI-based GTV delineation in NSCLC, laying the foundation for future exploration of MRI-based RT planning and guidance.

## 2 Methodology

### 2.1 Image acquisition

Patients with LA NSCLC due to receive radical treatment with sequential or concurrent CRT or RT alone, were imaged, following written informed consent to local research ethics approved protocols, at two UK institutions.

All patients had a 10-phase 4D RT planning CT (RTP CT) with intravenous contrast as per standard practice, acquired using a CT scanner (Philips Big Bore, Medical Systems, Best, Netherlands). Patients were positioned with arms up, using a modified extended wing board (Civco Extended Wingboard with T-Grip Handle). If the tumour was above the carina, patients could be scanned arms down, at clinician discretion, using a five-point shell and a flat radiotherapy board. All patients had ^18^F-FDG PET-CT during their diagnostic investigations as per standard practice.

All patients had MR imaging on a 70 cm diameter cylindrical bore 1.5 T MRI scanner (Siemens Aera, Erlangen, Germany) with flat table overlay, with spine coil and anterior receive coil on support, within 5 days of the RTP CT. No contrast agent was administered. MRI sequences included: (1) 3D T_1_-weighted (T_1_w) radial fast gradient echo with fat suppression (T_1_w Radial GRE), (2) navigator-triggered T_2_-weighted (T_2_w) turbo (i.e. fast) spin echo (TSE) (T_2_w TSE non-fat-sat), and, one or both of, (3) T_2_w TSE with fat saturation (T_2_w TSE fat-sat) and/or (4) T_2_w TSE DIXON sequence, generating water-only and fat-only images [11], [19] (Supplementary Table 1S).

T_1_w Radial GRE data were used to generate 4D-MRI as described by Rank *et al* [20]. The 4D-CT and the 4D-MR images were processed to generate mid-position (MidP) images as described by Wolthaus *et al* [21]. Patient positioning and immobilisation were consistent between MR and CT scans. However, the PET-CT was acquired in the diagnostic position. The PET-CT (average), MidP CT, MidP T_1_w Radial GRE (MidP T_1_w MR) and additional MR images were rigidly co-registered at the level of the tumour. Data including anonymised images and radiotherapy structure sets can be obtained from the corresponding author upon reasonable request.

### 2.2 Contouring

Contouring was performed using ‘Big Brother’ software [22]*.* Where delineation involved CT and PET-CT, clinicians contoured the GTV on the MidP CT, with the PET-CT on a secondary window for guidance. Where delineation involved MRI, CT, and PET-CT, the clinicians contoured the GTV on the MidP T_1_w MR, with the CT, PET-CT and other MRI datasets displayed in a secondary window. Contouring was performed on transverse images, with sagittal and coronal images available for corroboration.

### 2.3 Training workshops

Two MR contouring training workshops were undertaken. An initial virtual workshop was attended by twelve thoracic radiation oncologists from eight international centres, during which eight cases were reviewed alongside an MR radiologist-led tutorial. A second, face-to-face, workshop attended by eleven radiation oncologists from seven international centres, incorporated a further tutorial followed by a contouring session. Participants were then provided with three additional cases, that were subsequently contoured and discussed with the MR radiologist.

### 2.4 Thoracic MRI GTV contouring study

Nine additional cases were provided. From their respective centres, ten radiation oncologists from seven international centres independently contoured GTVs on the nine cases. Firstly, the contouring was performed by the clinician on the MidP CT, with PET-CT in secondary window. Then, at least two weeks later, contouring was performed by each clinician with MR radiologist support, on the MidP T_1_w MR, with CT, PET-CT and other MRI datasets in a secondary window.

### 2.5 Contour assessment and recommendation document preparation

The median GTV surface was derived to form the ‘consensus GTV contours’ [22]. An international panel of 11 radiation oncologists, 5 MR radiologists, and 2 MR physicists from 9 international centres discussed the individual and consensus GTV contours for all 9 cases. The consensus contours formed the basis of our recommendations for GTV contouring in LA NSCLC. The recommendations were prepared and reviewed by 3 MR radiologists from 3 different institutions and a subsequent face-to-face workshop of 11 radiation oncologists and 1 MR radiologist permitted final document review.

## 3 Results

### 3.1 Thoracic MR image interpretation and challenges

Challenges relevant to thoracic MR contouring were noted and are provided in Table 1S in Supplementary Appendix.

### 3.2 Contouring of the primary lung tumour

Primary tumour image characteristics for each clinical scenario were identified to aid GTV contouring (Comprehensive summary in Supplementary Table 1S).

#### 3.2.1 Discerning parenchymal changes around the primary tumour and areas of atelectasis

**Fig. 1:**
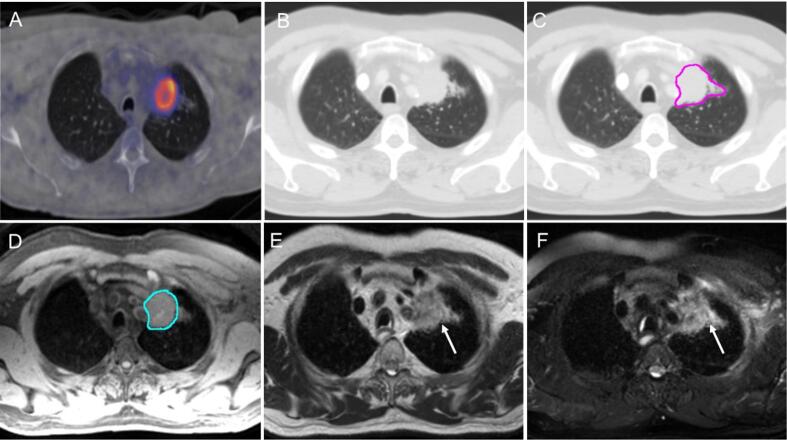
Parenchymal changes around the primary tumour; (A) PET-CT scan, (B) CT in lung windows (L: −474 HU, W: 1500 HU) without CT contour, (C) CT in lung windows with CT consensus contour (pink), (D) fat-suppressed T_1_w Radial GRE with MR consensus contour (light blue), (E) T_2_w TSE non-fat-sat demonstrating difference of signal intensity between tumour and atelectatic changes (arrow), (F) T_2_w TSE fat-sat demonstrating change of signal intensity (arrow). Signal intensity on the left side of the arrow was not included in the GTV as it was thought to represent adjacent atelectatic lung. (For interpretation of the references to colour in this figure legend, the reader is referred to the web version of this article.)

Discrepancies between CT and MR consensus contours were identified at the tumour/air interface, including areas of atelectasis and/or consolidation (Fig. 1C, 1D). It was agreed that signal intensity differences present on MR can represent the boundary between tumour and other non-malignant parenchymal changes (T_2_w fat-sat images; Fig. 1F). This is a useful principle, as the addition of MRI to CT and PET-CT may permit appropriately reduced GTVs in such scenarios.

#### 3.2.2 Tumour abutting, but not invading the chest wall

**Fig. 2:**
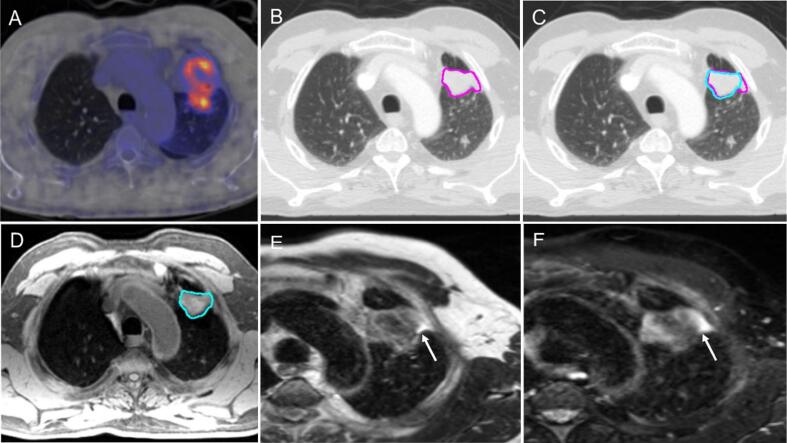
Tumour abutting the chest wall; (A) PET-CT scan, (B) CT in lung windows (L: −474 HU, W: 1500 HU) with CT consensus contour (pink), (C) CT scan in lung windows, with CT consensus contours (pink) and MR consensus contours (light blue), demonstrating more generous CT consensus contour at the chest wall/tumour interface; (D) fat-suppressed T_1_w Radial GRE with MR consensus contour (light blue), (E) T_2_w TSE non-fat-sat, (F) T_2_w TSE fat-sat. Arrows demonstrate difference in signal intensity between tumour and parenchymal/ pleural changes. (For interpretation of the references to colour in this figure legend, the reader is referred to the web version of this article.)

Tumour abutting the chest wall may induce a pleural reaction and/or parenchymal changes. The CT consensus GTV contour included these areas. On MRI, fluid content within a pleural reaction may display high T_2_w signal (Fig. 2E, 2F, Supplementary Fig. 4S). The panel agreed that such areas should not be included in the GTV as it does not represent macroscopic tumour. The panel discussed including this in the Clinical Target Volume (CTV) as microscopic disease and noted that motion of such tumours in relation to the neighbouring chest wall may provide information with regards to possible chest wall invasion.

#### 3.2.3 Tumour with invasion to chest wall/vertebrae

**Fig. 3:**
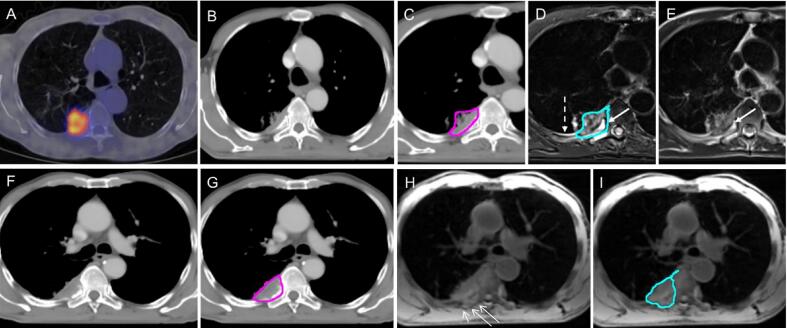
Chest wall invasion; (A) PET-CT scan, (B) CT scan in mediastinal windows (L: 46, W: 600 HU) showing no obvious cortical destruction of bone (C) CT scan with CT consensus contour (pink), (D) T_2_w TSE Dixon Water with hyperintensity of the rib (arrow). The MR consensus contour (light blue) reflects the choice of the clinicians to include the posterior rib in the GTV. There is also pleural reaction extending along the chest wall (broken arrow) and not included in the contour, (E) T_2_w TSE non-fat-sat image showing hyperintensity of the rib (arrow), (F) CT scan now showing cortical invasion, (G) CT scan showing inclusion of the involved bone within the CT consensus contour (pink), (H) T_1_w Radial GRE showing deeper invasion into posterior structures (thin arrows), (I) Fat-suppressed T_1_w Radial GRE with MR consensus contour (light blue) demonstrating slightly more generous volume of the MR consensus contour posteriorly. Although on the same slice on CT (E and G), cortical involvement was evident, and hence appropriately included in the GTV the MR images suggested deeper invasion included in the MR consensus contour. (For interpretation of the references to colour in this figure legend, the reader is referred to the web version of this article.)

Where the tumour invades the chest wall, ribs or vertebrae, changes in signal intensity may be apparent within the involved bone (Fig. 3D, 3E). It is difficult to determine whether this represents direct invasion of the tumour, bone marrow infiltration or oedema. In this context, cortical destruction visualised on CT, would confirm invasion (Fig. 3B, 3F).

Bone marrow may appear abnormal on MRI due to fat being replaced by water, without evidence of cortical destruction. These changes may display an intermediate signal intensity on T_1_w images and a high signal on the T_2_w imaging. Comparison to normal adjacent or contralateral ribs may facilitate interpretation. Consensus was reached that if the marrow appears abnormal on MRI where the tumour is suspected to invade bone, the bone should be included in the GTV (Fig. 3D).

The panel agreed that if there are abnormalities on adjacent ribs on MRI, the intervening intercostal muscles should be included within the GTV. The intercostal muscles may display high signal on T_2_w sequences, due to oedema caused by denervation of the muscles (due to tumour ingrowth) in the sub-acute phase. The panel discussed that alternatively intercostal muscles could be included in the CTV. This will need future pathological correlation studies to inform decisions on macroscopic or microscopic involvement.

A ‘tail’ of high T_2_w signal intensity may appear along the chest wall due to a pleural reaction. It is important to distinguish where this is due to pleural reaction or possible bone invasion/oedema (Fig. 3D). The panel agreed that the high-signal ‘tail’ should not be included in the GTV, as this may extend over a large area around the chest wall, making the GTV very large, whilst the likelihood of malignant involvement is low based on anatomical reasoning.

MRI allows visualisation of tissue extension into the chest wall. T_1_w images can demonstrate disruption of the normal soft tissue planes (Fig. 3H and 3I) and T_2_w images may provide additional information, such as muscle invasion depicted by high signal intensity. The T_2_w fat-suppressed images can display high contrast between tumour and normal soft tissue facilitating identification of the tumour. However, the T_2_w fat-suppressed images may also display high signal within *peri*-tumoural soft tissue which is due to oedema or inflammation and does not necessarily represent tumour infiltration.

#### 3.2.4 Intravascular invasion/ tumour invasion of pulmonary vessels

**Fig. 4:**
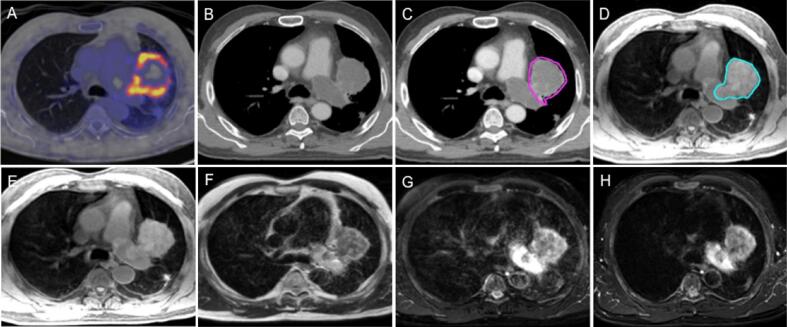
Intravascular invasion; (A – H): Same transverse slice showing invasion into the pulmonary artery and intravascular thrombus, (A) PET-CT scan, (B) CT scan (L: 46, W:600 HU) (C) CT scan with CT consensus contour (pink), (D) fat-suppressed T_1_w Radial GRE with MR consensus contour (light blue), (E) T_1_w Radial GRE, (F) T_2_ TSE non-fat-sat, (G) T_2_w TSE fat-sat, (H) T_2_ TSE Dixon Water. (For interpretation of the references to colour in this figure legend, the reader is referred to the web version of this article.)

There was noticeable difference between CT and MR consensus contours (Fig. 4). On MRI, the intra-arterial tumour and the associated thrombus were well distinguished. Whereas on CT, disease and thrombus appeared as filling defects of similar densities. Here, the panel agreed that the addition of MRI permitted improved visualisation of the disease.

#### 3.2.5 Primary tumour in continuation with atelectasis/collapsed lung

**Fig. 5:**
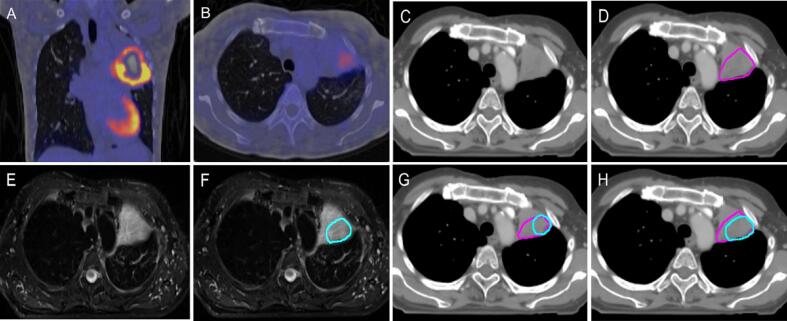
Atelectasis and lung collapse; (A) Coronal image of PET-CT scan showing left apical collapse, (B – F): Same transverse slice at the superior end of the tumour on (B) PET-CT, (C) CT scan (L: 46, W:600 HU) with no CT median contour (D) CT scan with CT median contour (pink), (E) T_2_w TSE fat-sat image without MR median contour, (F) T_2_w TSE fat-sat image with smaller MR median contour (light blue). T_2_w TSE fat-sat image demonstrates more tumour heterogeneity (E) compared to CT (C), although the panel agreed that the edge of the GTV was still challenging to define. (G – H): The two consecutive most apical slices of the tumour on CT scan (L: 46, W:600 HU), (G) is the most superior slice, followed by one consecutive slice (H) moving caudally, showing CT consensus contour (pink) and smaller MR consensus contour (light blue) with more generous CT consensus contour compared to MR consensus contour. (For interpretation of the references to colour in this figure legend, the reader is referred to the web version of this article.)

Defining the collapsed lung/tumour was challenging with both modalities. The CT consensus contour was more generous than for MR, especially at the most caudal part of the GTV (Fig. 5).

MR signal heterogeneity within the tumour, especially for T_2_w imaging, may support tumour identification within areas of collapsed lung. The panel agreed that in cases of atelectasis, all MRI sequences, and planes, should be reviewed and interpreted in conjunction with the PET-CT to assist in GTV delineation.

#### 3.2.6 Superior sulcus tumour

**Fig. 6:**
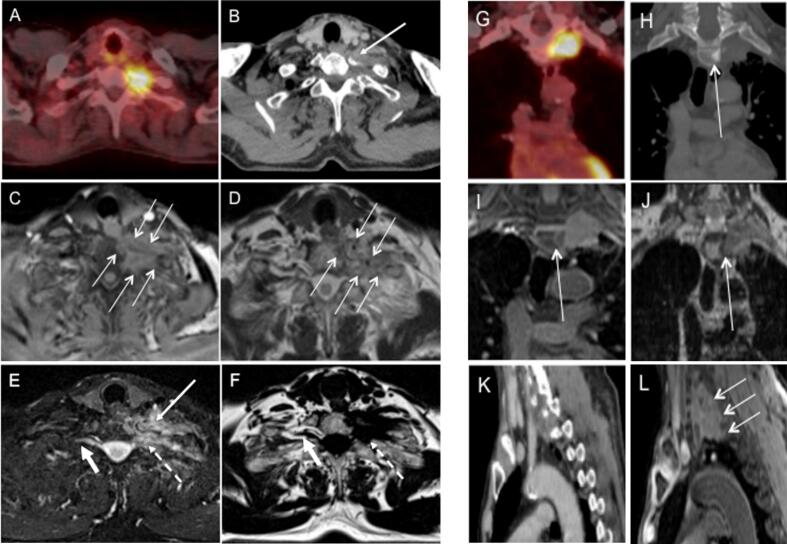
Superior sulcus tumour; (A – F). Same transverse slice of Pancoast tumour on (A) PET-CT scan, (B) CT scan (L: 46, W:600 HU) demonstrating cortical destruction (arrow), (C) fat-suppressed T_1_w Radial GRE demonstrating edges of the tumour (arrows), (D) T_2_w TSE non-fat-sat demonstrating tumour appearing darker than surrounding fat and edges of the tumour (arrows), (E) T_2_w TSE Dixon Water demonstrating tumour invasion of brachial plexus. Normal brachial plexus nerve root appears hyperintense (thick arrow). There is disruption of normal anatomy on the involved contralateral side (broken arrow) and associated hyperintense oedema (arrow) (F) T_2_w TSE Dixon Fat demonstrating tumour invasion of brachial plexus. Normal brachial plexus nerve root appears hypointense (thick arrow). There is disruption of normal anatomy on the involved contralateral side (broken arrow). (G – J): same coronal slice of a Pancoast tumour on (G) PET-CT, (H) CT scan in bone window (L:300, W:1500) showing mild hyperintensity in bone (arrow) but no obvious cortical destruction, (I) fat-suppressed T_1_w Radial GRE showing superior soft tissue extension of tumour and bone changes within the vertebral body (arrow), (J) T_2_w TSE non-fat-sat image with bone changes within the vertebral body (arrow) more pronounced than when compared to CT scan, (K and L): sagittal image on (K) CT scan showing suggestion of extension of the tumour in the intervertebral foramina and (L) fat-suppressed T_1_w Radial GRE showing more pronounced posterior extension of the tumour into the intervertebral foramina (arrows).

Superior sulcus tumours may invade the apical chest wall, vertebrae and brachial plexus. Assessment of all imaging planes is advised (Fig. 6 and Supplementary Fig. 1S).

On T_1_w images the tumour has higher signal intensity than surrounding bone and muscle, whilst on T_2_w non-fat-sat sequences the tumour has lower signal than fat-containing apical structures. These sequences demonstrate the disruption of the soft tissue planes. Tumour shows high signal intensity on the T_2_w fat-suppressed images, providing contrast between tumour and normal tissue.

On T_1_w images, the vessel lumen and wall appear hyperintense compared to the mediastinum. For T_2_w, the lumen appears black and the vessel wall displays a slightly higher signal. Patency or encasement of the vessels is assessed with MRI (Supplementary Fig. 1S).

Oedema within apical structures is displayed with T_2_w high signal (Fig. 6E) but, as for chest wall invasion, there can be uncertainty whether hyperintense areas represent direct tumour invasion or partly inflammation/oedema. The panel agreed that together the CT, PET-CT and MRI may support assessment of invasion, and that histopathologic correlation is needed to inform decisions of whether such changes should be included in GTV or CTV.

CT is useful for identifying cortical bone involvement (Fig. 6B). In some instances, however, there may be signal change within the bone on MRI, but none, or less, on CT (Fig. 6G–6J and Supplementary Fig. S1). The panel agreed they would include this abnormal area within the vertebrae in the GTV. Again, pathological correlation will aid decisions relating to GTV/ CTV delineation.

Brachial plexus involvement is best assessed on the T_2_w Dixon Water and T_2_w Dixon Fat by demonstrating disruption of the normal signal of the nerve roots (Fig. 6E and 6F).

### 3.3 MRI interpretation and contouring of the lymph nodes

#### 3.3.1 Supraclavicular fossa (SCF) lymph nodes (stations 1R/L)

SCF lymph nodes were well-visualised on transverse and coronal planes. On T_1_w images, nodes appear hyperintense compared to surrounding tissue. On the T_2_w Dixon water images, nodes appear hyperintense against the fat-supressed background. MRI allows inspection of the integrity of the supraclavicular vasculature (Supplementary Fig. S2).

#### 3.3.2 Upper mediastinal lymph nodes (stations 2R/L, 3A, 3P and 4R/L)

On T_1_w images these nodes appear hyperintense compared to the darker mediastinal fat. In the T_2_w non-fat-sat they appear dark within a brighter non-fat suppressed mediastinum, and on the T_2_w fat-sat/ T_2_w Dixon Water images, they are bright, similar in signal to the primary tumour (Supplementary Figs. S3-S6).

Structures within the mediastinum are susceptible to poor fat suppression and must be acknowledged. On T_1_w, T_2_w fat-sat and T_2_w Dixon Water, one would expect that lymph nodes are brighter compared to the fat suppressed mediastinum (which should appear dark), but they can appear darker than the brighter mediastinum (Table 1S and Supplementary Figs. 3S, 4S and 6S).

For the lymph nodes close to the oesophagus (3P, 4 L), the T_2_w non-fat-sat was useful in defining the boundary between lymph node(s) and oesophageal wall and improving GTV definition (Supplementary Fig. 5S).

There was good consistency between CT and MR consensus contours. In some cases, there was no generation of one of the consensus contours (CT or MR) indicating that not enough clinicians contoured the specific lymph node (on CT or MR). This was apparent with stations 2R and 3A (Supplementary Fig. 3S).

#### 3.3.3 Aortic lymph nodes (stations 5 and 6)

There was good agreement between CT and MR consensus contours. Lymph nodes are hyperintense compared to the mediastinal fat in the T_1_w, T_2_w fat-sat and Dixon water-only images. On T_2_w non-fat-sat, they are hypointense compared to the mediastinum (Supplementary Figs. 7S).

#### 3.3.4 Subcarinal nodes (station 7) and paraoesophageal lymph nodes (station 8)

Station 7 and 8 lymph nodes are hyperintense on T_1_w and T_2_w fat-sat compared with mediastinal fat. The T_2_w non-fat-sat sequence was the most useful in distinguishing the lymph node/oesophageal wall boundary (Supplementary Fig. 8S).

#### 3.3.5 Hilar lymph nodes (stations 10/11)

There was good agreement between the consensus CT and MR contours. MRI allows identification of the interface between hilar lymph nodes and vessels, and between hilar lymph nodes and mediastinal fat.

On T_1_w images, vascular lumen and wall appear hyperintense compared to the mediastinum. On T_2_w imaging, the lumen appears black, and hilar lymph nodes appear brighter relative to the lumen (Supplementary Fig. 10S). Hilar lymph nodes adjacent to mediastinal fat are hypointense on the T_2_w non-fat-sat compared to bright mediastinal fat (Supplementary Fig. 10S).

## 4 Discussion

This work describes the development of the first recommendations for GTV contouring in LA NSCLC on thoracic MRI. The document was formed through expert consensus, clinician training and feedback from two international contouring workshops.

The results highlighted differences in GTV contours when MRI was introduced. Importantly, in certain cases the MR consensus contour resulted in inclusion of additional structures. For example, in the case of chest wall invasion the MR GTV consensus contour included bone (ribs). In other cases, the MR consensus contour excluded areas considered non-malignant, such as *peri*-tumoural atelectasis. These observations suggest that MRI may assist with improved GTV definition in certain cases.

In cases of tumour proximity to bone such as ribs or vertebrae, MRI, especially T_2_w imaging allowed visualisation of bone marrow changes. Although, CT may allow visualisation of cortical disruption, it has less sensitivity for bone marrow changes than MRI [1]. With respect to deeper tissue invasion, MRI has been recognised for defining the extent of chest wall and superior sulcus tumour invasion [1], [2], [3], [4], [5], [6]. MRI findings in superior sulcus tumours, have been confirmed on pathological specimens [1], [2], [5] and have shown greater accuracy for identifying invasion than CT [5]. Differences between CT and MR consensus contours in the case of vascular invasion were evident; with intravascular thrombus supporting the usefulness of MRI to assess vascular integrity in superior sulcus tumours and SCF disease [1], [2], [3].

For lymph nodes, there was good consistency of CT and MR consensus contours, although uncertainty remained in some cases involving upper mediastinal nodes on both modalities. Karki *et al* noted larger IOV in lymph node contouring on MRI [24]. In our work, T_2_w non-fat-sat imaging was helpful in defining the lymph node/oesophageal wall boundary. However, poor fat suppression in the mediastinum affected lymph node visualisation, making delineation challenging. Further scenarios where MR generated some uncertainty, included:

1. Signal differences around the pleura due to proximity/invasion of tumour to chest wall.
2. Signal differences within bone in the absence of obvious cortical destruction which may represent bone marrow involvement or oedema.
3. Signal differences surrounding an invading tumour, (e.g superior sulcus tumours)

The panel agreed that for such areas, future systematic histopathologic correlation is needed to inform GTV/ CTV definition. Motion information may assist GTV definition. It has been shown that respiratory dynamic MRI with 2D cine images increases the accuracy of defining chest wall invasion by assessing mass shift of the tumour in relation to the chest wall [26].

Few studies have investigated MRI-based GTV delineation for lung cancer [25], [23], [26]. These studies are heterogeneous in methodology, MRI sequences, radiologist input and clinician training. Some suggest improved IOV with MRI [27], others show no impact or larger volumes when using MRI [28]. MRI has had limited use, even for diagnostic purposes, in thoracic malignancies. Hence, we felt that the first crucial steps, prior to exploring potential benefits, were clinician education and consensus recommendations for GTV contouring on thoracic MRI.

We acknowledge some limitations of this work. Firstly, with respect to the use of diagnostic PET-CT scans, with some patients having their PET-CT prior to chemotherapy and not at the time of the planning CT and MRI. However, in the UK, this reflects the ‘real life’ practice. Standardisation of PET-CT practice across the thoracic community will further enhance the potential of multimodality imaging to improve RT precision [30]. In addition, this study is qualitative and consensus-based and does not incorporate objective measures to quantify improvement. However, using the proposed recommendations, we are investigating interobserver variability in GTV contouring using MRI compared with standard of care CT and PET–CT. Nine patient cases were included in the study presented; all were scanned in the UK. This may not fully capture the variability of tumour presentations across international institutions. However, every effort was taken by the clinicians involved in the study to identify nine cases covering a wide range of clinical presentation and we believe that this is sufficient for these recommendations, that can be supplemented by the international community. These recommendations have not been validated against pathological specimens; rather, they are based on an extensive review conducted by senior international thoracic oncologists and radiologists. They are intended to provide a foundation for standardised contouring, thereby enabling systematic evaluation of MRI within the radiotherapy workflow and subsequent assessment of its impact on patient outcomes through improved target delineation. We anticipate that these recommendations will be beneficial to others, especially for initial and ongoing implementation of MR into lung RT planning and MRgRT [29]. The MR images acquired for this work did not require gadolinium-based contrast agents (GBCAs). The reason for this was to ensure applicability to repeated imaging, for example for MRgRT. However, the authors acknowledge the potential benefit of the inclusion of GBCAs to aid visualisation and contouring guidance for primary and nodal lesions [30], [31], [32], and this is worthy of further investigation in the context of radiotherapy workflows. Furthermore, the inclusion of diffusion weighted imaging (DWI) techniques to aid differentiation between lesion and atelectasis could prove beneficial but was not included in this work [33], [34].

It is acknowledged that FDG-PET remains the current standard for target volume delineation. However, MRI offers the potential for additional useful information to be integrated into radiotherapy and adaptive planning workflows by enabling, for example, even better differentiation between tumour and atelectasis, and treatment adaption based on tumour regression, or even biological changes, during the course of treatment, as well as accounting for uncertainties due to, for example, motion [11], [29]. However, caution is warranted when adapting treatment based on such findings in the absence of pathological validation, for example in the presence of subclinical disease.

In summary, GTV contouring on MRI revealed important topographical differences when compared to CT imaging, for specific clinical scenarios. We present a first set of practical recommendations for GTV contouring in LA NSCLC using thoracic MRI. These recommendations will require ongoing refinement as clinician training and experience increase. Further research is needed to assess observer variability, reproducibility of MR-based GTV contouring, and histopathologic validation. As with the integration of PET-CT in lung RT planning, future work should explore the clinical impact of MR for RT planning and the role of MRgRT within prospective clinical trials for patients with LA NSCLC.

## CRediT authorship contribution statement

**Anna-Maria Shiarli:** Conceptualization, Data curation, Formal analysis, Investigation, Methodology, Project administration, Writing – original draft, Writing – review & editing. **Michael J. Dubec:** Conceptualization, Data curation, Formal analysis, Investigation, Methodology, Project administration, Writing – original draft, Writing – review & editing. **Merina Ahmed:** Investigation, Methodology, Writing – review & editing. **Jon T. Asmussen:** Investigation, Methodology, Writing – review & editing. **Hannah Bainbridge:** Investigation, Methodology, Writing – review & editing. **José S. Belderbos:** Investigation, Methodology, Writing – review & editing. **Sean Brown:** Investigation, Methodology, Writing – review & editing. **Johan Bussink:** Investigation, Methodology, Writing – review & editing. **David Cobben:** Investigation, Methodology, Writing – review & editing. **Bram H.J. Geurts:** Investigation, Methodology, Writing – review & editing. **Andrew Hope:** Investigation, Methodology, Writing – review & editing. **John Kavanagh:** Investigation, Methodology, Writing – review & editing. **Dow-Mu Koh:** Investigation, Methodology, Writing – review & editing. **Ferry Lalezari:** Investigation, Methodology, Writing – review & editing. **Alexander V. Louie:** Investigation, Methodology, Writing – review & editing. **Laura G. Merckel:** Investigation, Methodology, Writing – review & editing. **Firdaus A.M. Hoesein:** Investigation, Methodology, Writing – review & editing. **James P.B. O’Connor:** Investigation, Methodology, Writing – review & editing. **Rocio Perez-Johnston:** Investigation, Methodology, Writing – review & editing. **Tyson J. Reeve:** Investigation, Methodology, Writing – review & editing. **Andreas Rimner:** Investigation, Methodology, Writing – review & editing. **Peter S.N. van Rossum:** Investigation, Methodology, Writing – review & editing. **Dominic A.X. Schinagl:** Investigation, Methodology, Writing – review & editing. **Tine Schytte:** Investigation, Methodology, Writing – review & editing. **Craig Stevens:** Investigation, Methodology, Writing – review & editing. **Alex Tan:** Investigation, Methodology, Writing – review & editing. **Rob H.N. Tijssen:** Investigation, Methodology, Writing – review & editing. **Nina Tunariu:** Investigation, Methodology, Writing – review & editing. **Marcel Van Herk:** Conceptualization, Data curation, Formal analysis, Investigation, Methodology, Project administration, Resources, Software, Supervision, Visualization, Writing – original draft, Writing – review & editing. **Joost J.C. Verhoeff:** Investigation, Methodology, Writing – review & editing. **Andreas Wetscherek:** Conceptualization, Investigation, Methodology, Resources, Software, Visualization, Writing – review & editing. **Rianne Wittenberg:** Investigation, Methodology, Writing – review & editing. **David Woolf:** Investigation, Methodology, Writing – review & editing. **Corinne Faivre-Finn:** Conceptualization, Data curation, Funding acquisition, Investigation, Methodology, Project administration, Resources, Supervision, Visualization, Writing – original draft, Writing – review & editing. **Fiona McDonald:** Conceptualization, Formal analysis, Funding acquisition, Investigation, Methodology, Project administration, Resources, Supervision, Visualization, Writing – original draft, Writing – review & editing.

## Declaration of competing interest

The authors declare that they have no known competing financial interests or personal relationships that could have appeared to influence the work reported in this paper.
