# Supplementary material for: Recommendations for contouring of gross tumour volume for locally advanced lung cancer using magnetic resonance imaging
Source: Phys Imaging Radiat Oncol. 2026 Mar 15;38:100948. doi: 10.1016/j.phro.2026.100948 (PMC13019103; doi:10.1016/j.phro.2026.100948)
Supplement: Supplementary Data 1 [file mmc1.docx]

**Supplementary Material**


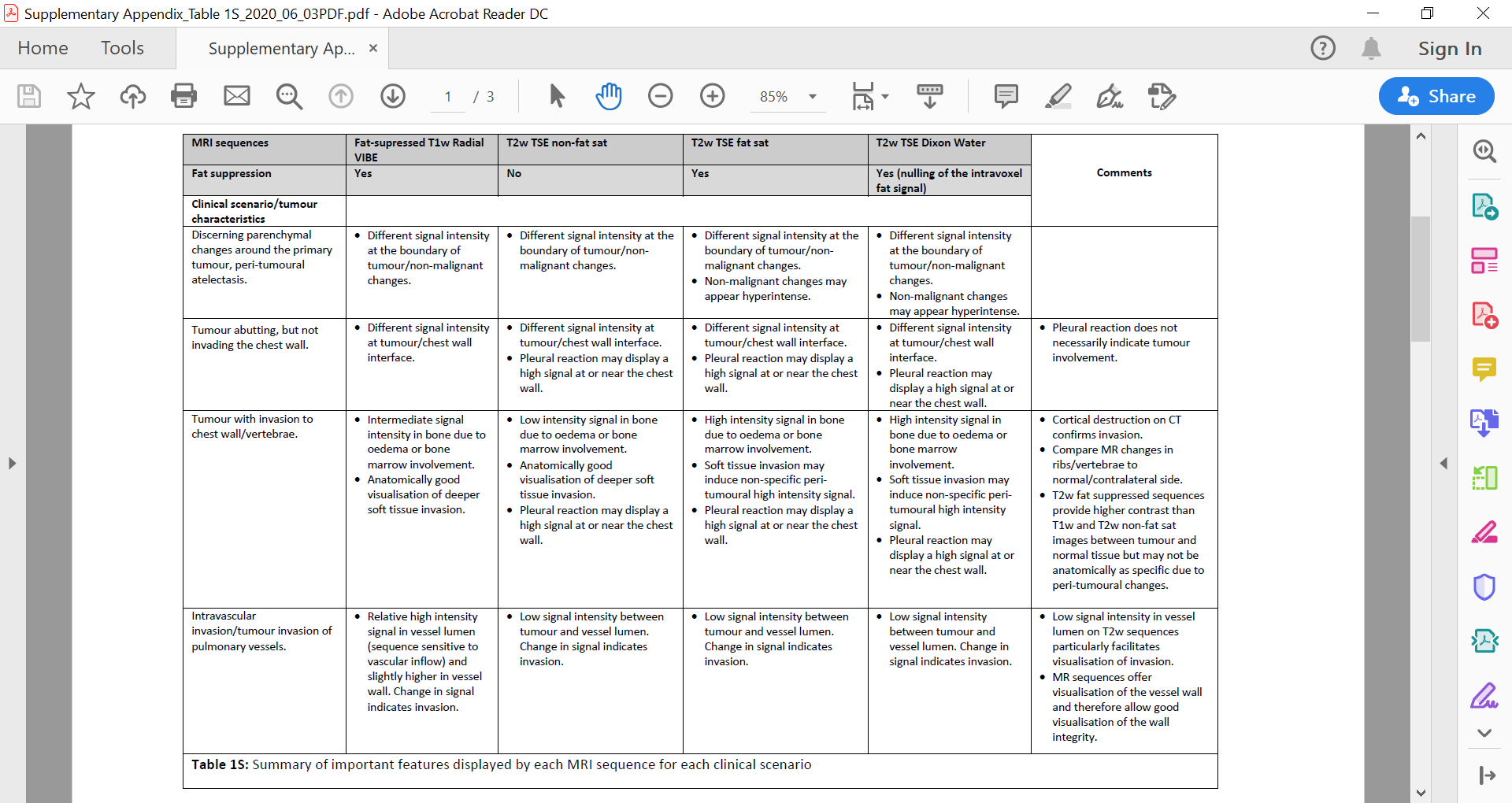


*T_1_w Radial VIBE = T_1_w Radial Gradient Echo (GRE)


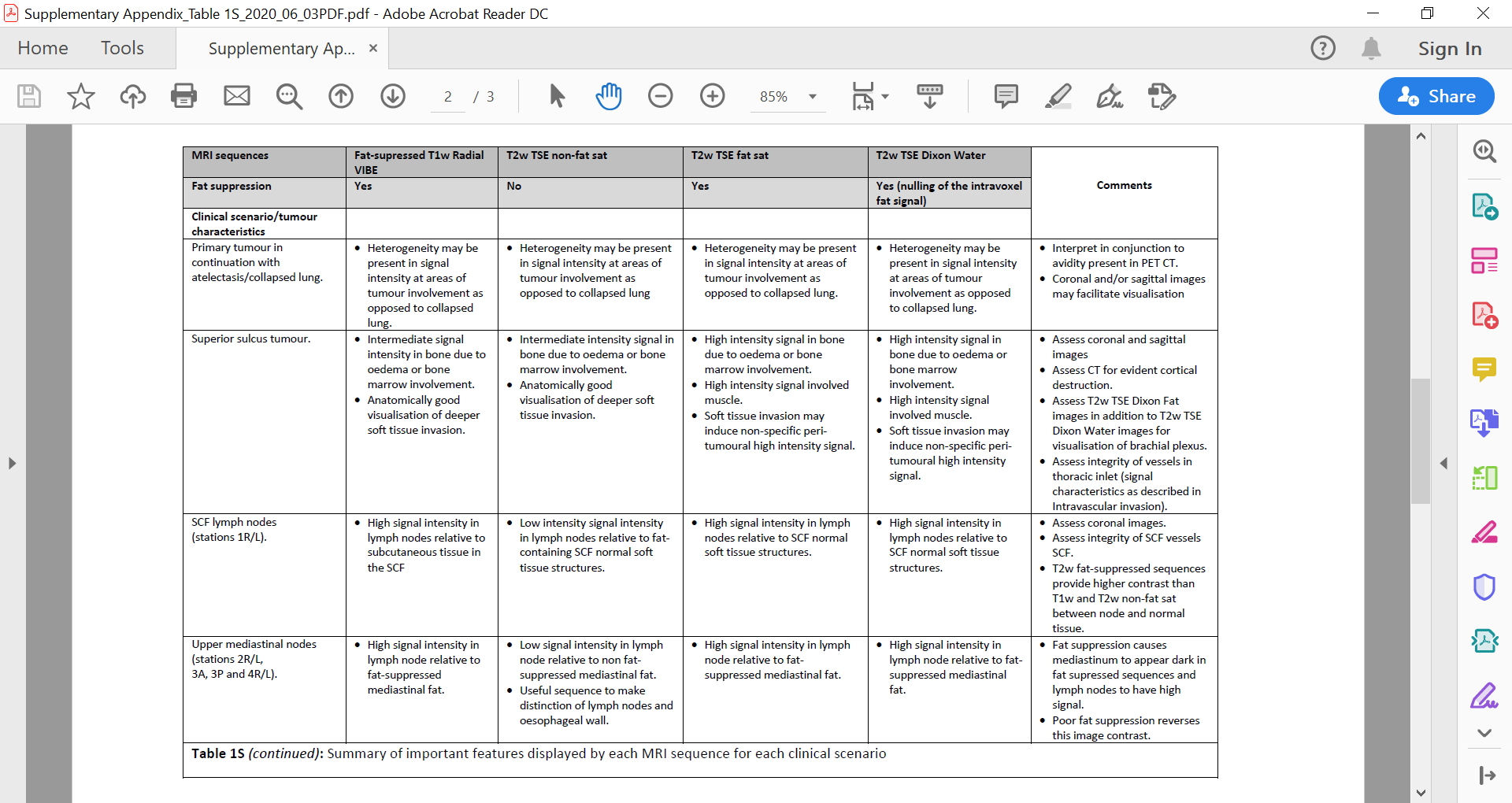


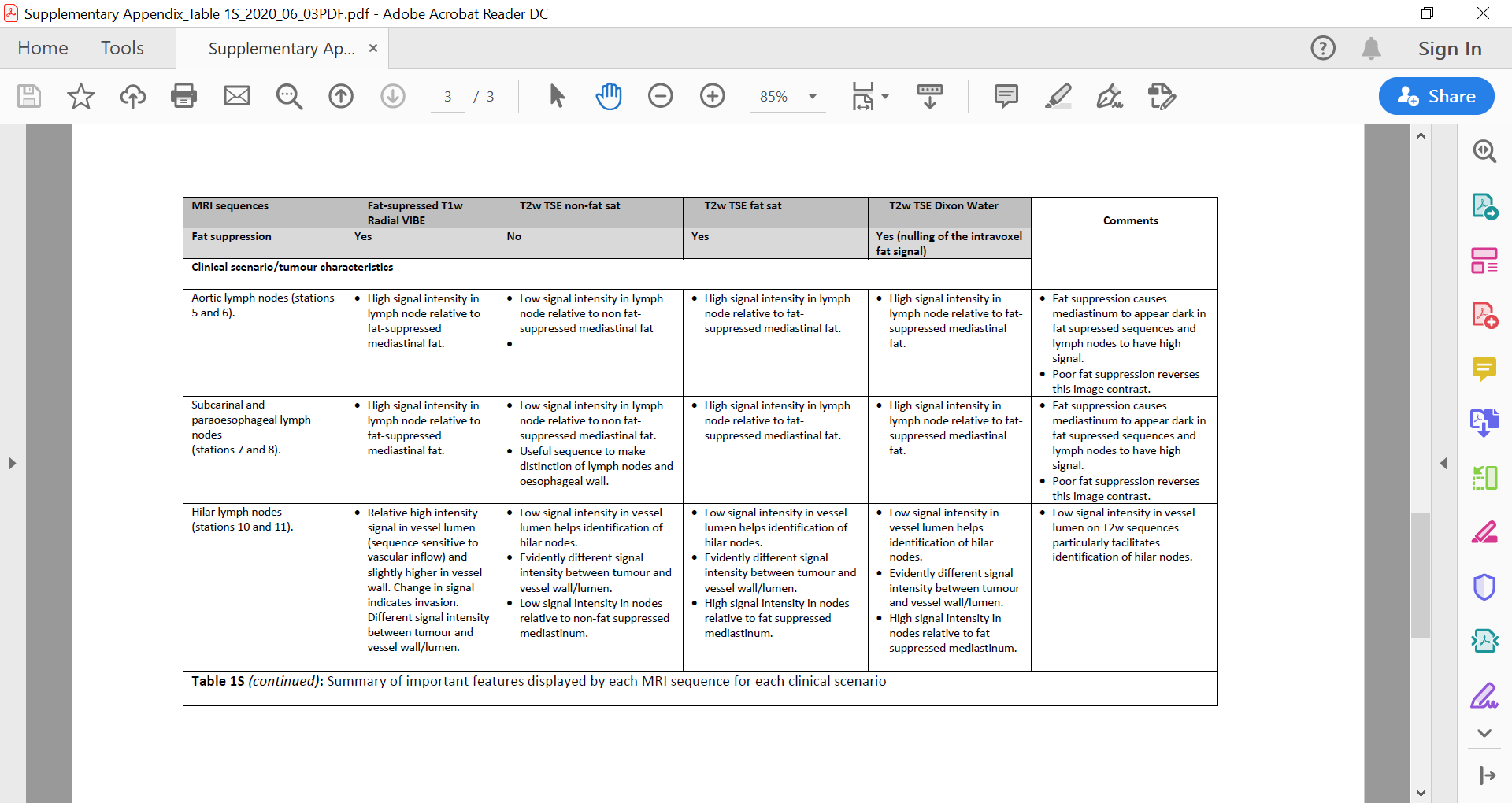


**
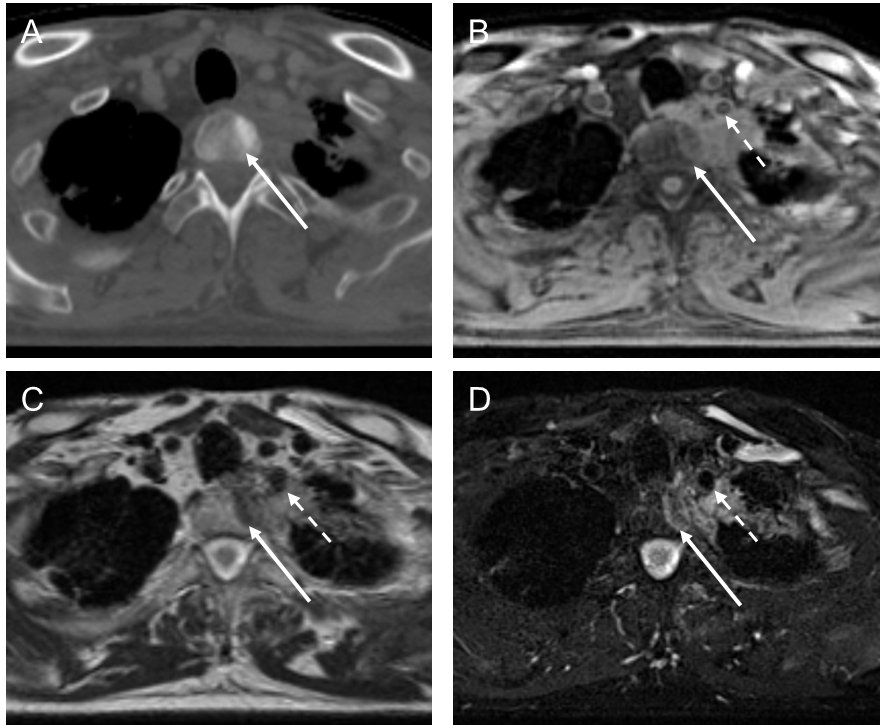
**

**Figure 1S**: **A-D** same axial slice of a Pancoast tumour showing bone changes (arrows) on **A:** CT in bone window (L: -474 HU, W: 1500 HU), **B:** T1w Radial GRE, **C:** T2w TSE non-fat sat, **D:** T2w TSE Dixon Water. Patency of subclavian artery demonstrated on MR images (broken arrows).


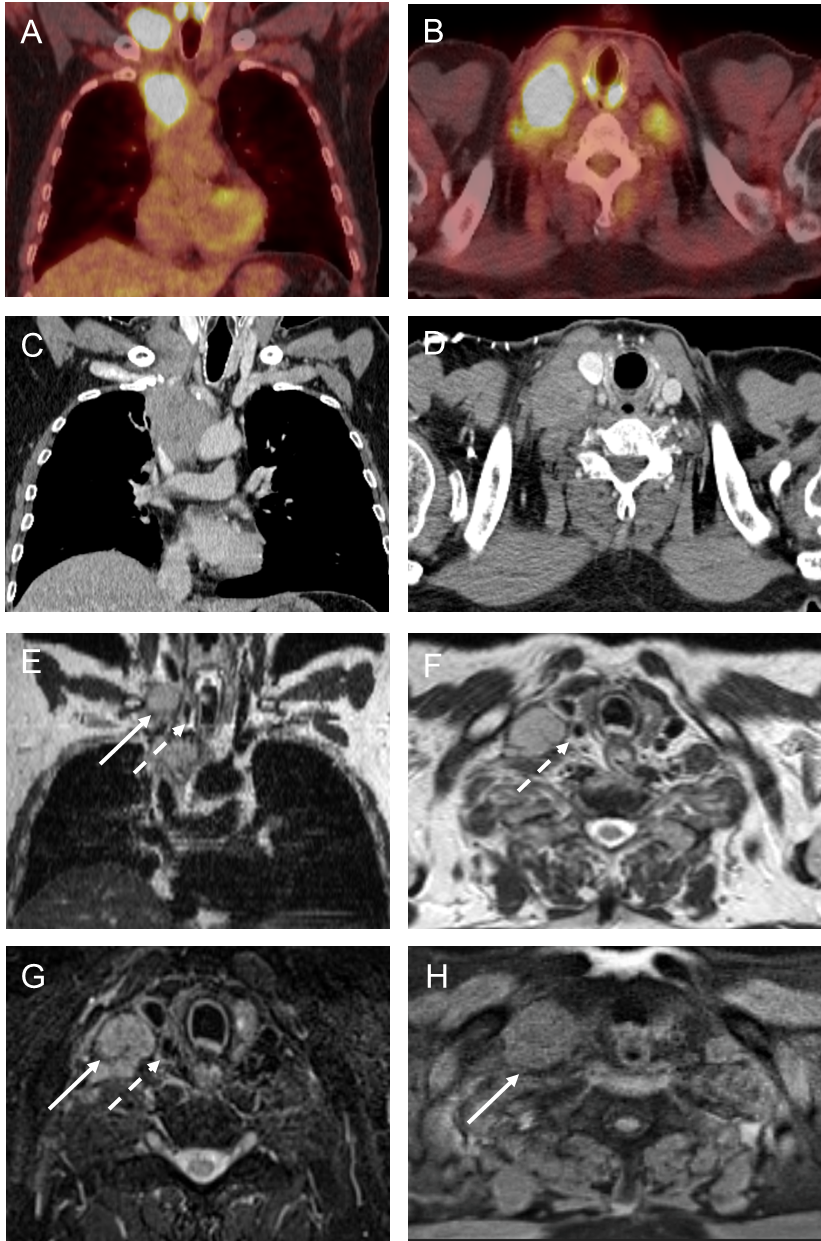


**Figure 2S:** Coronal and axial images of the same patient demonstrating an SCF (1R) lymph node (arrows). **A:** PET CT coronal image demonstrating avid SCF (1R) node and ipsilateral 2R nodes, **B:** PET CT axial image across avid SCF (1R) node, **C:** CT coronal image of SCF (1R) and 2R node (L: 46, W:600 HU), **D:** CT axial image of SCF 1R node, **E:** T2w TSE non-fat sat coronal image of SCF (1R) node, **F:** T2w TSE non-fat sat axial image across SCF (1R) node, **G:** T2w TSE Dixon Water axial slice across SCF (1R) node, **H:** Fat suppressed T1w Radial GRE axial slice across SCF (1R) node. Patency of subclavian artery demonstrated by identification of vessel wall and lumen on MR images (broken arrows).


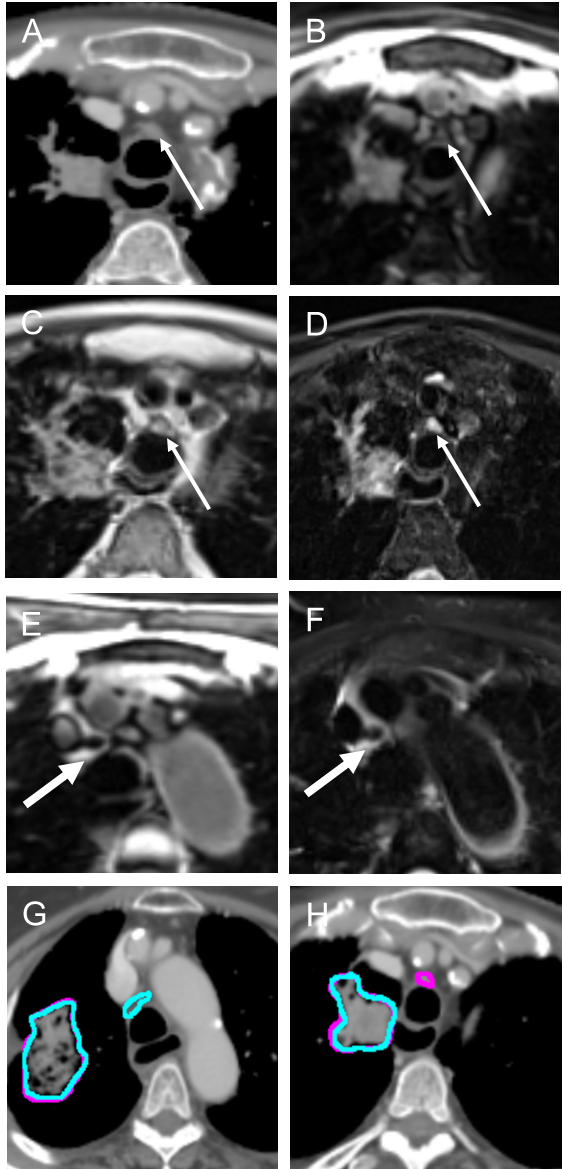


**Figure 3S: A-D** same axial slice of station 2 (2R) lymph nodes (thin arrows). These not avid on PET CT but positive EBUS, **A:** CT scan (L: 46, W: 600 HU), **B:** T1w Radial GRE where the lymph node has higher signal than surrounding mediastinum **C:** T2w TSE non-fat sat where the lymph node has lower signal than the brighter mediastinal fat, **D:** T2w TSE Dixon Water with lymph node having high signal intensity, similar to the primary tumour. **E-F:** 2R lymph nodes demonstrating failure of fat suppression (thick arrows) on fat-suppressed T1w Radial GRE **(E)** and T2w fat sat **(F). G-H:** Consensus contours generated for 2R lymph nodes in two cases **G:** CT consensus contour (pink) and MR consensus contour (light blue) shown on CT scan (L: 46, W: 600 HU) demonstrating only MR consensus contour present in this case for 2R lymph node; **H:** CT consensus contour and MR consensus contour on CT scan (L: 46, W: 600 HU) demonstrating only CT consensus contour generated in this case for 2R lymph node.


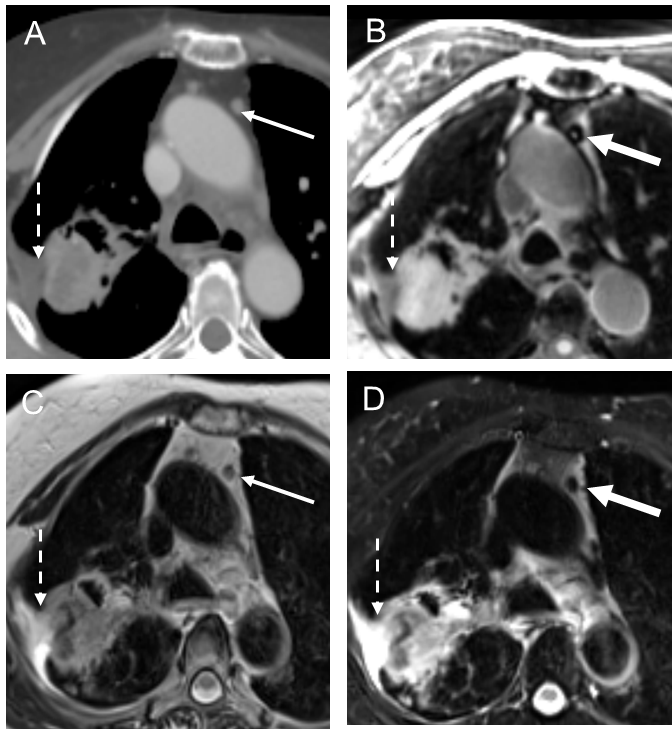


**Figure 4S:** 3A upper mediastinal lymph nodes, **A:** CT scan (L: 46, W: 600 HU) demonstrating 3A lymph nodes (arrow), **B:** Fat suppressed T1w Radial GRE with failure of fat suppression showing hypointense lymph node (thick arrow) **C:** T2w TSE non-fat sat showing well demarcated low signal lymph nodes within mediastinal fat (arrow), **D:** T2w TSE fat-sat showing failure of fat suppression hence low signal in lymph node (thick arrow). Also notice change in signal intensity between tumour and chest wall relating to pleural reaction/atelectatic changes as mentioned for the primary tumour abutting chest wall.


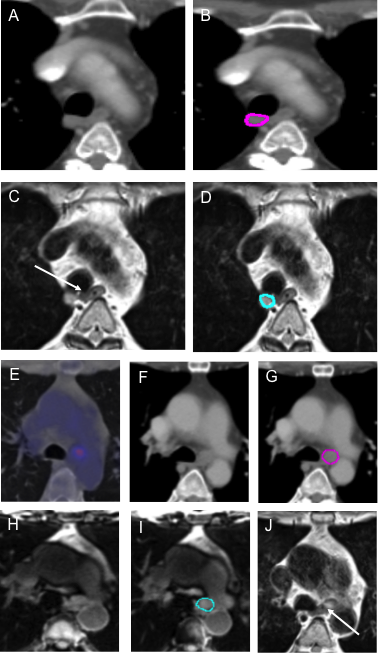


**Figure 5S: A-D:** Prevertebral lymph nodes (3P) **A:** CT scan (L: 46, W:600 HU), **B:** CT scan with CT consensus contour (pink), **C:** T2w TSE non-fat sat image showing good distinction between lymph node and oesophageal wall (arrow) which also appears hypointense compared to the mediastinal fat , **D:** T2w TSE non-fat sat image with MR consensus contour (light blue); **E-J:** 4L lymph nodes on, **E:** PET CT scan, **F:** CT scan (L: 46, W:600 HU), **G:** CT scan with CT consensus contour, **H:** fat suppressed T1w Radial GRE with MR consensus contour, **I:** fat-suppressed T1w Radial GRE without contour, **J:** T2w TSE non-fat sat with lymph node appearing darker than darker than brighter mediastinal fat and demonstrating clear distinction from the oesophageal wall (arrow).


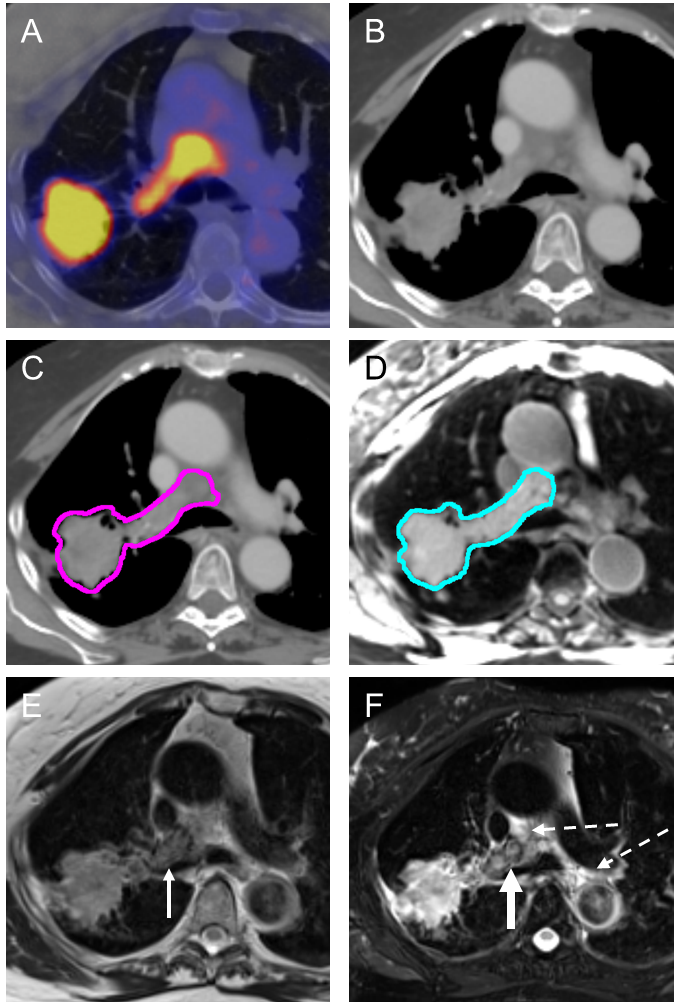


**Figure 6S:** 4R lymph nodes on **A:** PET CT scan, **B:** CT scan (L: 46, W:600 HU), **C:** CT scan with CT consensus contour (pink), **D:** fat-suppressed T1w Radial GRE with MR consensus contour (light blue) and 4R lymph node appearing hyperintense compared to mediastinum and similar intensity to tumour, **E:** T2w TSE non-fat sat demonstrating lower signal in lymph nodes (arrow) compared to fatty mediastinum, **F:** T2w TSE fat sat image demonstrating lower signal in the lymph node (thick arrow) than the brighter mediastinum due to failure of fat suppression (dashed arrows).


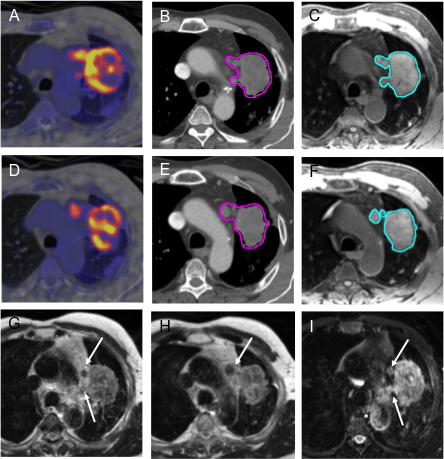


**Figure 7S: A-C:** Sub-aortic lymph nodes (station 5) on **A:** PET CT, **B:** CT scan (L: 46, W:600 HU) with CT consensus contour (pink), **C:** fat-suppressed T1w Radial GRE with MR consensus contour (light blue); **D-F:** Para-aortic lymph nodes (station 6) on **D:** PET CT, **E:** CT scan (L: 46, W:600 HU) with CT consensus contours (pink), **F:** T1w Radial GRE with MR consensus contour (light blue); **G – H:** T2w TSE non-fat sat demonstrating aortic lymph nodes (arrows); station 5 **(G)** and station 6 **(H)**, **I:** T2w TSE fat sat demonstrating station 5 lymph nodes (arrows).


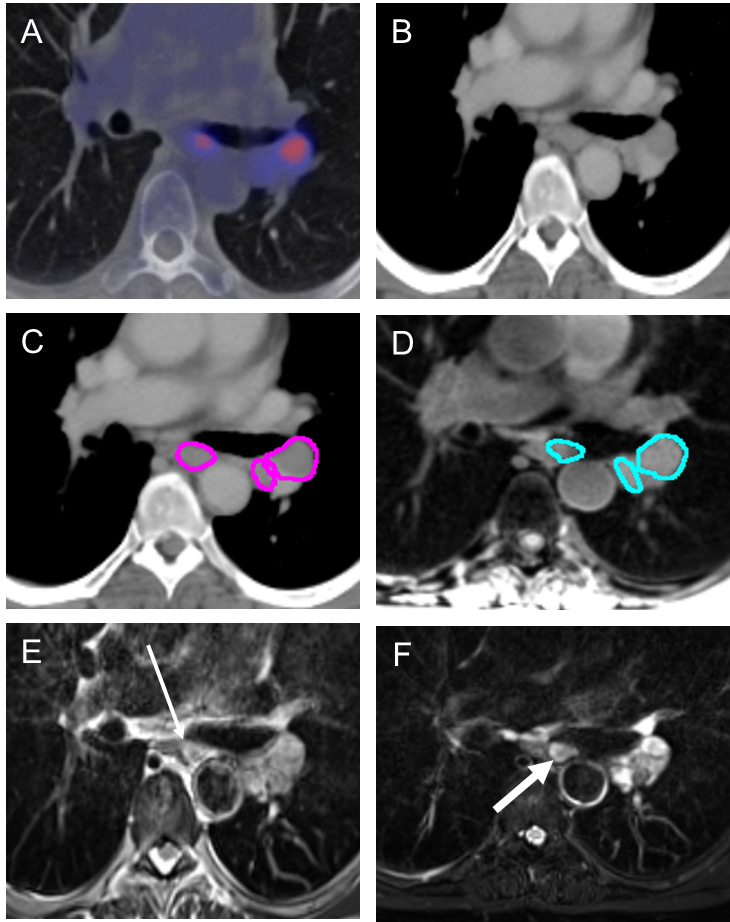


**Figure 8S:** Subcarinal lymph node on **A:** PET CT scan, **B:** CT scan (L: 46, W: 600 HU), **C:** CT scan with CT consensus contour (pink), **D:** fat-suppressed T1w Radial GRE with MR consensus contour (light blue), **E:** T2w TSE non-fat sat sequence allows distinction between lymph node and oesophageal wall (arrow), **F:** T2w TSE fat sat sequence demonstrating higher signal in subcarinal lymph node compared to the darker mediastinum.

**
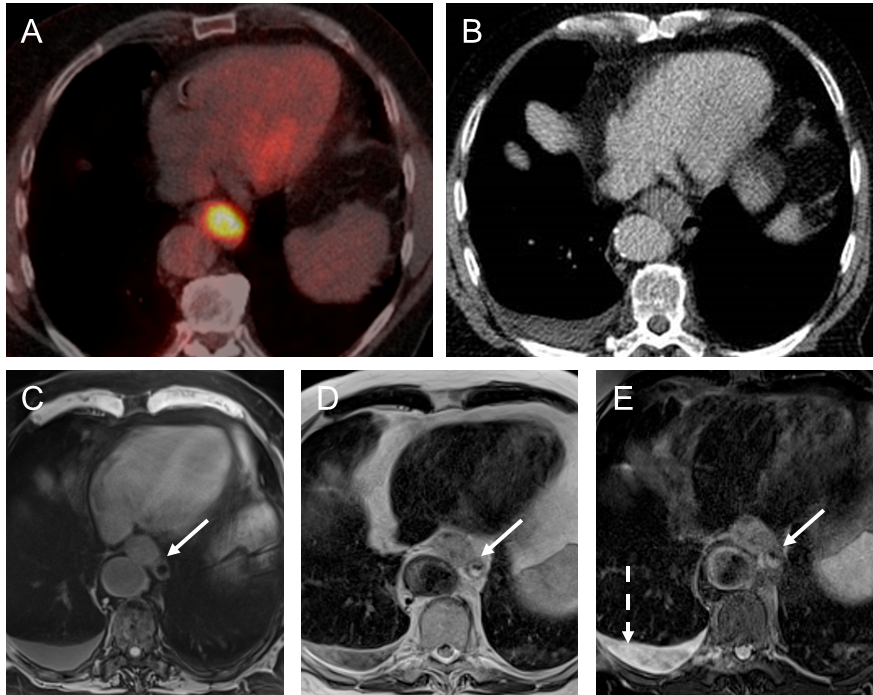
**

**Figure 9S:** Paraoesophageal lymph node on **A:** PET CT, **B:** CT scan (L: 46, W: 600 HU), **C:** fat-suppressed T1w Radial GRE demonstrating hyperintense node within darker mediastinum, **D:** T2w TSE non-fat sat demonstrating the node and oesophagus appearing darker than mediastinum, **D:** T2w fat sat demonstrating hyperintensity within the lymph node appearing brighter. Distinction demonstrated between oesophageal wall and lymph node on MR images (arrows). Right sided pleural effusion demonstrated appearing mostly hyperintense in the T2w fat sat image due to the presence of fluid (broken arrow).

**
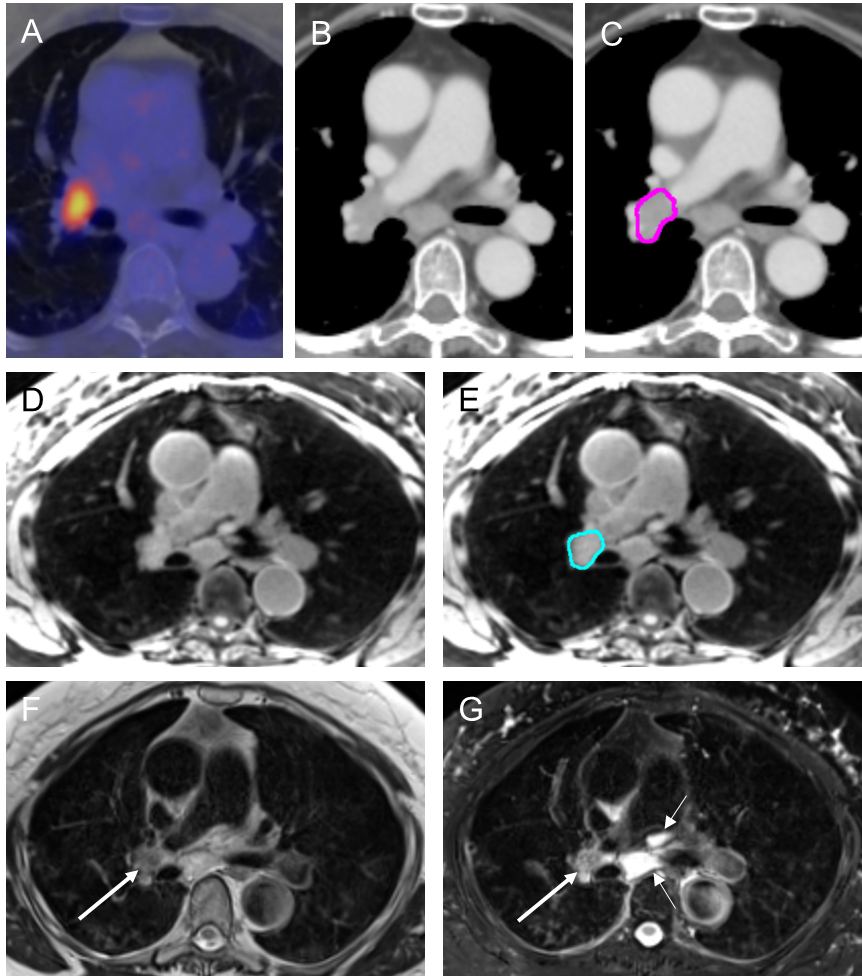
**

**Figure 10S:** Hilar lymph nodes (station 10) on **A:** PET CT scan, **B:** CT scan (L: 46, W:600 HU) without consensus contour, **C:** CT scan with CT consensus contour (pink), **D:** T1w Radial GRE without consensus contour, **E:** T1w Radial GRE with MR consensus contour (light blue), **F:** T2w TSE non-fat sat demonstrating hypointense lymph node (arrow) compared to mediastinal fat, **G:** T2w TSE fat sat with failure of fat suppression, therefore the mediastinal areas (small arrows) appear even brighter than the involved lymph node (arrow).
